# Supplementary material for: 28-day all-cause mortality and associated factors in cancer patients with bacteremia at a peruvian referral center
Source: PLoS One. 2026 May 22;21(5):e0349381. doi: 10.1371/journal.pone.0349381 (PMC13196976; doi:10.1371/journal.pone.0349381)
Supplement: S1 File — Additional methodological data and analyses, including sensitivity analyses for 48-hour and 28-day all-cause mortality. (PDF) [file pone.0349381.s001.pdf]

Supplemental Table 1. Variables included in the bivariate analysis for 48-hour all-cause mortality.

Variable	p-value*	Inclusion in the multivariable analysis
Age	0.4555	YES
Sex	0.559	YES
Active cancer	0.710	NO
Advanced neoplasm	0.090	YES
Cancer stage	0.057	NO
Metastasis	0.024	YES
Metachronous neoplasm	0.782	NO
Polymicrobial bacteremia	1.000	NO
Enterococcus faecalis	0.607	NO
Staphylococcus aureus	1.000	NO
Coagulase-negative staphylococci (CoNS)	1.000	NO
Acinetobacter baumannii	0.044	YES
Escherichia coli	0.487	NO
Klebsiella pneumoniae	1.000	NO
Pseudomonas aeruginosa	0.411	NO
Empirical antibiotic therapy	0.543	NO
Adequate empirical antibiotic therapy	0.011	YES
Adequate empirical antibiotic therapy	0.076	YES
ICU admission	0.005	NO

Acute kidney injury	0.038	YES
Altered mental status	0.001	NO
Fever	0.005	NO
Sepsis	0.000	NO
Septic shock	1.000	NO
Respiratory failure at admission	0.000	YES
Leukocyte count	0.1103	NO
Neutrophil count	0.0735	NO
Band neutrophils	0.1074	NO
Platelet count	0.0152	NO
Hemoglobin	0.9376	NO
Mechanical ventilation	0.005	YES
Prior hospitalization	0.884	NO
Prior antibiotic use	0.288	NO
Charlson Comorbidity Index	0.3286	NO
Prior major surgery	0.235	NO
Chemotherapy	0.690	NO
Radiotherapy	1.000	NO
Corticosteroid	1.000	NO
Hormonal therapy	0.241	NO
Immunosuppressive therapy	0.390	NO
Neutropenia (by grades)	0.008	YES

Body Mass Index (categorical)	0.616	NO
Solid tumor vs hematological malignancy	1.000	NO
Fluoroquinolone-resistant Enterobacterales	0.419	NO
Multidrug-resistant Enterobacteriaceae	0.820	NO
Extensively drug-resistant Enterobacterales	0.333	NO
AmpC-producing pathogens	0.710	NO
Methicillin-resistant CoNS	0.649	NO
Presence of resistant profile	0.661	NO
Gram-positive bacteria	0.797	NO
Gram-negative bacteria	1.000	NO
Multidrug-resistant Gram-negative bacteria	0.447	NO
Extensively drug-resistant Gram-negative bacteria	1.000	NO
Nosocomial bacteremia	0.785	NO
Enterobacteriaceae	0.516	NO
Extended-spectrum β-lactamase-producing Enterobacteriaceae	0.249	NO
Non-fermenting Gram-negative bacteria	0.344	NO
Extended-spectrum β-lactamase-producing Gram-negative bacteria	0.221	NO
Carbapenem-resistant Gram-negative bacteria	0.232	NO
Neutropenia	0.015	NO
Gastrointestinal malignancy	0.345	NO
Gynecologic malignancy	0.401	NO

Genitourinary malignancy	1.000	NO
Male reproductive malignancy	0.145	NO
Hematologic malignancy	1.000	NO
Lung malignancy	0.084	NO
Empirical antibiotic monotherapy	0.000	YES
Number of antibiotic classes received (4 groups)	0.001	NO
Primary source bacteremia	0.000	YES
Urinary source	0.002	NO
Pulmonary source	1.000	NO
Coagulopathy	0.076	YES
Leukopenia/Leukocytosis	0.002	YES
Left shift	1.000	NO
Anemia	0.529	NO

*Chi-square test or Fisher's exact test were used for categorical variables. After assessing normality with the Shapiro-Wilk test, non-categorical variables were analyzed using the t-test or the Mann-Whitney U test.

Supplemental Table 2. Variables included in the bivariate analysis for 28-day all-cause mortality.

Variable	p-value*	Inclusion in multivariable analysis
Age	0.2391	YES
Sex	0.279	YES
Type of cancer (by organ system)	0.380	NO
Active cancer	0.091	YES
Advanced neoplasm	0.006	YES
Cancer stage	0.006	YES
Metastasis	0.000	YES
Metachronous neoplasm	0.979	NO
Polymicrobial bacteremia	0.033	YES
Enterococcus faecalis	0.512	NO
Staphylococcus aureus	1.000	NO
Coagulase-negative Staphylococci (CoNS)	0.002	YES
Acinetobacter baumannii	0.099	YES
Escherichia coli	0.412	NO
Klebsiella pneumoniae	0.779	NO
Pseudomonas aeruginosa	0.432	NO
Empirical antibiotic therapy	1	NO
Adequate empirical antibiotic therapy	0.586	NO
Place of bacteremia acquisition	0.135	YES

ICU admission	0.023	YES
ICU admission within 48 hours	0.094	NO
Source of bacteremia	0.000	YES
Persistent bacteremia	0.017	YES
Atrial fibrillation	0.891	NO
Acute kidney injury	0.219	NO
Body mass index (continuous)	0.1599	NO
Altered mental status	0.000	NO
Fever	0.000	NO
Tachycardia	0.000	NO
Hypotension	0.000	NO
Respiratory rate	0.000	NO
Sepsis	0.000	YES
Septic shock	0.000	YES
Respiratory failure at admission	0.000	NO
Respiratory failure	0.000	YES
Leukocyte count	0.4479	NO
Neutrophil count	0.6231	NO
Band neutrophils (left shift)	0.4946	NO
Platelet count	0.0002	NO
Prior major surgery	0.599	NO
Central venous catheter	0.099	NO

Peripherally inserted central catheter (PICC)	0.099	NO
Port-a-Cath	0.550	NO
Urinary catheter	0.005	NO
Dialysis catheter	1.000	NO
Parenteral nutrition	1.000	NO
Nephrostomy tube	1.000	NO
Biliary catheter	0.670	NO
Mechanical ventilation	0.000	YES
Prior hospitalization	0.167	YES
Prior antibiotic use	0.445	NO
Number of antibiotics received	0.343	NO
Charlson Comorbidity Index	0.0003	YES
Prior major surgery	0.693	NO
Chemotherapy	0.143	YES
Radiotherapy	0.978	NO
Corticosteroid	0.257	NO
Biological agents	0.758	NO
Tyrosine kinase inhibitors	0.300	NO
Immunobiological agents	0.332	NO
Hormonal therapy	0.054	YES
Immunosuppressive therapy	0.548	NO
Neutropenia (by grades)	0.155	YES

Body Mass Index (categorical)	0.064	NO
Solid tumor vs hematological malignancy	0.440	NO
Fluoroquinolone-resistant Enterobacteriaceae	0.287	NO
Multidrug-resistant Enterobacteriaceae	0.347	NO
Extensively drug-resistant Enterobacteriaceae	0.797	NO
AmpC-producing pathogens	0.438	NO
Methicillin-resistant CoNS	0.007	YES
Presence of resistant profile	0.669	NO
Gram-positive bacteria	0.039	YES
Gram-negative bacteria	0.120	YES
Multidrug-resistant Gram-negative bacteria	0.301	NO
Extensively drug-resistant Gram-negative bacteria	0.766	NO
Nosocomial bacteremia	0.588	NO
Enterobacteriaceae	0.303	NO
Extended-spectrum β-lactamase-producing Enterobacteriaceae	0.397	NO
Non-fermenting Gram-negative bacilli	0.766	NO
Extended-spectrum β-lactamase-producing Gram-negative bacteria	0.456	NO
Difficult-to-treat resistant Gram-negative bacteria	0.332	NO
Carbapenem-resistant Gram-negative bacteria	1	NO
Severe neutropenia	0.109	YES
Neutropenia	0.139	NO

Gastrointestinal malignancy	0.613	NO
Gynecological malignancy	0.413	NO
Genitourinary malignancy	0.978	NO
Male reproductive malignancy	0.176	YES
Hematologic malignancy	0.440	NO
Pulmonary malignancy	0.333	NO
Empirical antibiotic monotherapy	0.395	NO
Number of antibiotics received (4 groups)	0.320	NO
Secondary source of bacteremia	0.000	NO
Primary bacteremia	0.001	NO
Urinary source	0.000	NO
Respiratory source	0.013	NO
Coagulopathy	0.008	YES
Leukopenia/Leukocytosis	0.004	YES
Left shift	0.014	YES
Anemia	0.865	NO

*Chi-square test or Fisher's exact test were used for categorical variables. After assessing normality with the Shapiro-Wilk test, non-categorical variables were analyzed using the t-test or the Mann-Whitney U test.

Supplemental Table 3. Empiric antibiotic therapy and 28-day all-cause mortality bivariate analysis*†

Antibiotic	Frequency	Alive	Deceased	p-value
Meropenem	173 (59.0)	116 (58.3)	57 (60.6)	0.703
Ceftriaxone	73 (24.9)	48 (24.1)	25 (26.6)	0.647
Cefepime	48 (16.4)	37 (18.6)	11 (11.7)	0.137
Piperacillin-Tazobactam	45 (15.4)	28 (14.1)	17 (18.1)	0.374
Metronidazol	24 (8.2)	16 (8.0)	8 (8.5)	0.891
Vancomycin	24 (8.2)	13 (6.5)	11 (11.7)	0.132
Ertapenem	21 (7.2)	20 (10.1)	1 (1.1)	0.005
Clindamycin	12 (4.1)	9 (4.5)	3 (3.2)	0.758
Ciprofloxacin	5 (1.7)	5 (2.5)	0 (0)	0.180
Oxacillin	5 (1.7)	4 (2.0)	1 (1.1)	1.000
Other	24 (8.2)			

*Calculated on 293 patients unless otherwise specified.
† Values are number (%).

Supplemental Table 4. Appropriate empirical therapy according to antibiotic usage*†

Antibiotic	Frequency	Appropriate	Inappropriate	p-value
Meropenem	173 (59.0)	161 (70.0)	12 (19.1)	<0.001
Ceftriaxone	73 (24.9)	53 (23.0)	30 (31.8)	0.157
Cefepime	48 (16.4)	34 (14.8)	14 (22.2)	0.157
Piperacillin-Tazobactam	45 (15.4)	36 (15.7)	9 (14.3)	0.790
Metronidazol	24 (8.2)	16 (7.0)	8 (12.7)	0.141
Vancomycin	24 (8.2)	20 (8.7)	4 (6.4)	0.547
Ertapenem	21 (7.2)	18 (7.8)	3 (4.8)	0.403
Clindamycin	12 (4.1)	11 (4.8)	1 (1.6)	0.257
Ciprofloxacin	5 (1.7)	4 (1.7)	1 (1.6)	0.934
Oxacillin	5 (1.7)	4 (1.7)	1 (1.6)	0.934
Other	24 (8.2)	-	-	-

*Calculated on 293 patients unless otherwise specified. Values are number (%) unless otherwise noted.
† Values are number (%).

Supplemental Table 5. Coagulase-Negative Staphylococci species*†

Bacteria	Frequency
Staphylococcus epidermidis	9 (42.9)
Staphylococcus hominis	7 (33.3)
Staphylococcus haemolyticus	2 (9.5)
Staphylococcus lugdunensis	1 (4.8)
Staphylococcus warneri	1 (4.8)
Staphylococcus capitis	1 (4.8)

*Calculated on 21 isolates.
†Values are number (%).

Supplemental Table 6. 28-day all-cause-mortality by bacterial species*†

Pathogen	Total	Deceased	p-value
Escherichia coli	138 (47.1)	41 (29.7)	0.412
Klebsiella pneumoniae	43 (14.7)	13 (30.2)	0.779
Coagulase-negative *Staphylococci*	21 (7.2)	13 (61.9)	0.002
Pseudomonas aeruginosa	20 (6.8)	8 (40.0)	0.432
Staphylococcus aureus	12 (4.1)	4 (33.3)	1.000
Enterococcus faecalis	11 (3.8)	2 (18.2)	0.512
Serratia marcescens	9 (3.1)	3 (33.3)	1.000
Enterobacter cloacae	8 (2.7)	2 (25.0)	1.000
Acinetobacter baumannii	4 (1.4)	3 (75.0)	0.099

*Calculated on 293 patients.
† Values are number (%).

Supplemental Table 7. Antimicrobial Resistance profiles and 28-day all-cause mortality*†

Phenotype	Total	Deceased	p-value
Gram-negative bacteria	245 (83.6)		
Carbapenem-resistant, n = 245	8 (3.3)	3 (37.5)	0.700
Multidrug-resistant, n = 245	100 (40.8)	36 (36.0)	0.101
Extensively drug-resistant, n = 245	34 (13.9)	12 (35.3)	0.486
Difficult-to-treat resistance, n = 245	5 (2.0)	3 (60.0)	0.164
Extended-spectrum β-lactamase-producing Enterobacteriaceae, n = 211	92 (43.6)	92 (28.3)	0.565
Gram-positive bacteria	52 (17.8)		
Methicillin-resistant coagulase-negative *Staphylococci*, n = 21	15 (71.4)	10 (66.7)	0.631

*Calculated on 293 patients unless otherwise specified.

† Values are number (%).

Supplemental Table 8. 28-day all-cause mortality according to the Charlson Comorbidity Index components*†

Component	Frequency	Alive	Deceased	p-value
Age				0.514
< 50 years	49 (16.7)	34 (17.1)	15 (16.0)	
50 - 59 years	56 (19.1)	40 (20.1)	16 (17.0)	
60 - 69 years	61 (20.8)	45 (22.6)	16 (17.0)	
70 - 79 years	70 (23.9)	46 (23.1)	24 (25.5)	
≥ 80 years	57 (19.4)	34 (17.1)	23 (24.5)	
Myocardial infarction	8 (2.7)	6 (3.0)	2 (2.1)	1.000
Heart failure	13 (4.4)	5 (2.5)	8 (8.5)	0.030
Peripheral vascular disease	15 (5.1)	11 (5.5)	4 (4.3)	0.781
Stroke	14 (4.8)	8 (4.0)	6 (6.8)	0.389
Dementia	8 (2.7)	6 (3.0)	2 (2.1)	1.000
Chronic pulmonary disease	51 (17.4)	33 (16.6)	18 (19.2)	0.589
Connective tissue disease	3 (1.0)	2 (1.0)	1 (1.1)	1.000
Peptic ulcer disease	2 (0.7)	1 (0.5)	1 (1.1)	1.000
Liver disease	14 (4.8)			0.313
Mild	7 (2.4)	3 (1.5)	4 (4.3)	
Moderate to severe	7 (2.4)	4 (2.0)	3 (3.2)	
Diabetes mellitus	50 (17.1)			0.862
Uncomplicated	45 (15.4)	30 (15.1)	15 (16.0)	
End-organ damage	5 (1.7)	3 (1.5)	2 (2.1)	
Hemiplegia	9 (3.1)	6 (3.0)	3 (3.2)	1.000
Moderate to severe CKD	20 (6.8)	13 (6.5)	7 (7.5)	0.772

Solid tumor	255 (87.0)			0.001
Localized	106 (36.2)	86 (43.2)	20 (21.3)	
Metastatic	149 (50.9)	88 (44.2)	61 (64.9)	
Leukemia	16 (5.5)	12 (6.0)	4 (4.3)	0.533
Lymphoma	23 (7.9)	13 (6.5)	10 (10.6)	0.223
AIDS	0 (0.00)	0 (0.00)	0.00	-

*Calculated on 293 patients.

† Values are number (%).

Supplemental Table 9. Sensitivity analysis restricted to individuals with solid tumors and monomicrobial bacteremia*†

	28 day-all-cause mortality		Bivariate	Multivariable‡	
**Characteristics**	**Alive (n = 167)**	**Deceased (n= 72)**	**p-value**	**RR§[95% CI‖]**	**p-value**
Charlson Comorbidity Index, mean ± SD	6.9 ± 2.7	8.44 ± 2.2	<0.001**	1.2 [1.1-1.3]	<0.001
Primary source of bacteremia	58 (34.7)	47 (65.3)	<0.001††	3.4 [1.8-6.5]	<0.001
Coagulase-negative *Staphylococci*	3 (1.8)	9 (12.5)	0.001††	2.0 [1.4-3.0]	<0.001
Coagulopathy	40 (24.2)	30 (42.3)	0.005††	1.6 [1.2-2.2]	0.005
Sepsis	60 (35.9)	56 (77.8)	<0.001††	3.9 [1.8-5.6]	<0.001
Septic shock	37 (22.2)	39 (54.2)	<0.001††	5.9 [2.4-6.0]	<0.001

*Calculated on 239 patients.
†Values are number (%) unless otherwise noted.
‡Adjusted for age and sex.
§RR: relative risk.
‖CI: robust confidence interval.
SD: standard deviation.
**Student's *t*-test.
††Chi-square test.
