# Supplementary material for: 28-day all-cause mortality and associated factors in cancer patients with bacteremia at a peruvian referral center
Source: PLoS One. 2026 May 22;21(5):e0349381. doi: 10.1371/journal.pone.0349381 (PMC13196976; doi:10.1371/journal.pone.0349381)
Supplement: Attachment — Submitted filename: PONE-D-25-64720_reviewer_ON.docx [file pone.0349381.s003.docx]

PLOS One

28-Day All-Cause Mortality and Related Factors in Cancer Patients with Bacteremia at a Peruvian Referral Center

--Manuscript Draft--

| Manuscript Number: | PONE-D-25-64720 |
| --- | --- |
| Article Type: | Research Article |
| Full Title: | 28-Day All-Cause Mortality and Related Factors in Cancer Patients with Bacteremia at a Peruvian Referral Center |
| Short Title: | 28-Day All-Cause Mortality and Related Factors in Cancer Patients with Bacteremia |
| Corresponding Author: | Yosué Vera, M.D.  Universidad Peruana Cayetano Heredia Facultad de Medicina Lima, PERU |
| Keywords: | Bacteremia; cancer; Mortality; antimicrobial resistance |
| Abstract: | Introduction: The global prevalence of cancer has increased in recent decades. Cancer patients are especially vulnerable to invasive infections like bacteremia. In Peru, the rising incidence of bloodstream infections caused by resistant pathogens has become a major public health issue. There is limited information about this complication in the Peruvian cancer population. No previous local studies have assessed mortality or explored the impact of antimicrobial resistance on patient outcomes in this group. Methods: This study aimed to evaluate 28-day all-cause mortality and its associated factors among cancer patients with bacteremia at a referral cancer center in Lima. We retrospectively analyzed data from first episodes of bacteremia in hospitalized adult patients between July 2020 and June 2024.  Results: A total of 293 patients were included in the study. The mean age was 65.42 ± 15.31 years, and 53.92% were female. Most patients had solid tumors (84.30%) and active disease (91.81%), with digestive cancers being the most common (34.13%). The 28-day all-cause mortality rate was 32.08%. Empirical antimicrobial therapy was appropriate in 80.70% of cases. Gram-negative bacteria (GNB) predominated  (82.80%), with Escherichia coli being the most frequently isolated pathogen (45.70%). Among Enterobacteriaceae, 43.60% were extended-spectrum beta-lactamase (ESBL) producers, and 3.27% of GNB were carbapenem-resistant. Multivariate Poisson regression identified the Charlson Comorbidity Index (RR 1.11; 95% CI 1.02–1.21), sepsis (RR 2.26; 95% CI 1.19–4.29), septic shock (RR 2.74; 95% CI 1.58–4.76), and respiratory failure (RR 1.66; 95% CI 1.05–2.62) as independent factors associated with increased 28-day mortality. A urinary source of infection was found to be associated with less mortality (RR 0.38; 95% CI 0.19–0.77).  Conclusion: At this cancer referral center, one-third of patients with bacteremia died within 28 days. Mortality was primarily caused by the severity of infection and specific comorbidities, rather than antimicrobial resistance. |
| Order of Authors: | Yosué Isaías Vera, MD |
|  | Nicolás Ismael Zamudio, MD |
|  | Bruno Eduardo Rojas, MD |
|  | Pedro Legua, MD |
|  | Brian Delfin, MD |
|  | Dany Rivera |
|  | Kathiuska Tutaya |
|  | Karol Villavicencio, RN |
|  | Angie Palomino, RN |
|  | Sissy Monsalve |
|  | Paola Montenegro |
|  |  |

|  | Ivan Aguilar, MD |
| --- | --- |
|  | Maribel Robles |
|  | Yenka La Rosa, MD |
|  | Giuliana Cardenas, MD |
|  | Frank Young, MD |
|  | Carlos Rafael Seas, MD, FIDSA |
| Additional Information: |  |
| Question | Response |
| Financial Disclosure  Enter a financial disclosure statement that describes the sources of funding for the work included in this submission. Review the [submission guidelines](http://journals.plos.org/plosone/s/submission-guidelines#loc-financial-disclosure-statement) for detailed requirements. View published research articles from [PLOS ONE](https://journals.plos.org/plosone/) for specific examples.  This statement is required for submission and will appear in the published article if the submission is accepted. Please make sure it is accurate. | The author(s) received no specific funding for this work. |
| Funded studies Enter a statement with the following details: - Initials of the authors who received each award - Grant numbers awarded to each author - The full name of each funder - URL of each funder website - Did the sponsors or funders play any role in the study design, data collection and analysis, decision to publish, or preparation of the manuscript? |  |
| Did you receive funding for this work? |  |
| Competing Interests  Use the instructions below to enter a competing interest statement for this submission. On behalf of all authors, disclose any [competing interests](http://journals.plos.org/plosone/s/competing-interests) that could be perceived to bias this work—acknowledging all financial support and any other relevant financial or nonfinancial competing interests. | The authors have declared that no competing interests exist. |

| This statement is required for submission and will appear in the published article if the submission is accepted. Please make sure it is accurate and that any funding sources listed in your Funding Information later in the submission form are also declared in your Financial Disclosure statement.  View published research articles from [PLOS ONE](http://journals.plos.org/plosone/) for specific examples. |  |
| --- | --- |
| NO authors have competing interests Enter: The authors have declared that no competing interests exist. Authors with competing interests Enter competing interest details beginning with this statement: I have read the journal's policy and the authors of this manuscript have the following competing interests: [insert competing interests here] |  |
| * typeset |  |
| Ethics Statement  Enter an ethics statement for this submission. This statement is required if the study involved:   - Human participants - Human specimens or tissue - Vertebrate animals or cephalopods - Vertebrate embryos or tissues - Field research   Write "N/A" if the submission does not require an ethics statement.  General guidance is provided below. Consult the [submission guidelines](http://journals.plos.org/plosone/s/submission-guidelines#loc-guidelines-for-specific-study-types) for detailed instructions. Make sure that all | The project was approved by the Institutional Research Ethics Committee of  Universidad Peruana Cayetano Heredia and Clínica Oncosalud. No access to patients' personal data was granted, as all information was coded for the study, ensuring that the authors had no access to participants’ identifying details. Data were collected retrospectively between August 1st and December 15th, 2024. Access to the database was restricted to the principal investigators. |

information entered here is included in the

Methods section of the manuscript.

Format for specific study types

Human Subject Research (involving human

participants and/or tissue)

Give the name of the institutional review

board or ethics committee that approved the

study

•

Include the approval number and/or a

statement indicating approval of this

research

•

Indicate the form of consent obtained

(

written/oral) or the reason that consent was

not obtained (e.g. the data were analyzed

anonymously)

•

Animal Research (involving vertebrate

animals, embryos or tissues)

Provide the name of the Institutional Animal

Care and Use Committee (IACUC) or other

relevant ethics board that reviewed the

study protocol, and indicate whether they

approved this research or granted a formal

waiver of ethical approval

•

Include an approval number if one was

obtained

•

If the study involved

non-human primates

,

add

additional details

about animal welfare

and steps taken to ameliorate suffering

•

If anesthesia, euthanasia, or any kind of

animal sacrifice is part of the study, include

briefly which substances and/or methods

were applied

•

Field Research

Include the following details if this study

involves the collection of plant, animal, or

other materials from a natural setting:

Field permit number

•

Name of the institution or relevant body that

granted permission

•

Data Availability

Authors are required to make all data

underlying the findings described fully

available, without restriction, and from the

time of publication. PLOS allows rare

exceptions to address legal and ethical

Yes - all data are fully available without restriction

| concerns. See the [PLOS Data Policy](http://journals.plos.org/plosone/s/data-availability) and [FAQ](http://journals.plos.org/plosone/s/data-availability#loc-faqs-for-data-policy) for detailed information.  A Data Availability Statement describing where the data can be found is required at submission. Your answers to this question constitute the Data Availability Statement and will be published in the article, if accepted. |  |
| --- | --- |
| Important: Stating ‘data available on request from the author’ is not sufficient. If your data are only available upon request, select ‘No’ for the first question and explain your exceptional situation in the text box. |  |
| Do the authors confirm that all data underlying the findings described in their manuscript are fully available without restriction? |  |
| Describe where the data may be found in full sentences. If you are copying our sample text, replace any instances of XXX with the appropriate details.   - If the data are held or will be held in a public repository, include URLs, accession numbers or DOIs. If this information will only be available after acceptance, indicate this by ticking the box below. For example: All XXX files   are available from the XXX database (accession number(s) XXX, XXX.).   - If the data are all contained within the manuscript and/or Supporting Information files, enter the following: All relevant data are within the manuscript and its Supporting Information files. - If neither of these applies but you are able to provide details of access elsewhere, with or without limitations, please do so. For example:   Data cannot be shared publicly because of [XXX]. Data are available from the XXX Institutional Data Access / Ethics Committee (contact via XXX) for researchers who meet the criteria for access to confidential data. | All relevant data are within the manuscript and its Supporting Information files. |

| The data underlying the results presented in the study are available from (include the name of the third party and contact information or URL).  • This text is appropriate if the data are owned by a third party and authors do not have permission to share the data.  * typeset |  |
| --- | --- |
| Additional data availability information: |  |

Manuscript	[Click here to access/download;Manuscript;Manuscript](https://www2.cloud.editorialmanager.com/pone/download.aspx?id=42972983&guid=043f2690-8fc2-4721-a90e-e6deb9af44f9&scheme=1)

[final.docx](https://www2.cloud.editorialmanager.com/pone/download.aspx?id=42972983&guid=043f2690-8fc2-4721-a90e-e6deb9af44f9&scheme=1)

1

2

3

4	**28-Day All-Cause Mortality and Related Factors in Cancer Patients with Bacteremia**

# 5 at a Peruvian Referral Center

6

7 Yosué I. Vera^1,3¶*^, MD , Nicolás I. Zamudio^1¶^, MD , Bruno E. Rojas, MD^1¶^, Pedro Legua^3,4&^, MD,
8 Brian Delfin^3&^, MD, Danny Rivera^2&^, Kathiuska Tutaya^2&^, Karol Villavicencio^3^, RN, Angie Palomino^3^,
9 RN, Sissy Monsalve^3^, RN, Paola Montenegro^3^, MD, Ivan Aguilar^3^, MD, Maribel Robles^3^, Yenka La 10	Rosa^3^, MD, Giuliana Cardenas^3^, MD, Frank Young^3^, MD, Carlos R. Seas^1,3,4¶^, MD, FIDSA.

11

12

13 ^1^ Facultad de Medicina Alberto Hurtado. Universidad Peruana Cayetano Heredia, Lima, Perú.
14 ^2^ Laboratorio de Microbiología, AUNA, Lima, Peru.
15 ^3^ Clínica Oncosalud, AUNA, Lima, Peru.
16 ^4^ Instituto de Medicina Tropical Alexander von Humboldt, Universidad Peruana Cayetano Heredia, 17	Lima, Peru.

18

19

20 ^*^ Corresponding author
21 E-mail: yosue.vera@upch.pe

22

23 ^¶^ These authors contributed equally to this work.
24 ^&^ These authors also contributed equally to this work.
25 Carlos R. Seas is a Senior Author

# Abstract

**Introduction:** The global prevalence of cancer has increased in recent decades. Cancer patients are especially vulnerable to invasive infections like bacteremia. In Peru, the rising incidence of bloodstream infections caused by resistant pathogens has become a major public health issue. There is limited information about this complication in the Peruvian cancer population. No previous local studies have assessed mortality or explored the impact of antimicrobial resistance on patient outcomes in this group.

**Methods:** This study aimed to evaluate 28-day all-cause mortality and its associated factors among cancer patients with bacteremia at a referral cancer center in Lima. We retrospectively analyzed data from first episodes of bacteremia in hospitalized adult patients between July 2020 and June 2024.

**Results:** A total of 293 patients were included in the study. The mean age was 65.42 ± 15.31 years, and 53.92% were female. Most patients had solid tumors (84.30%) and active disease (91.81%), with digestive cancers being the most common (34.13%). The 28-day all-cause mortality rate was 32.08%. Empirical antimicrobial therapy was appropriate in 80.70% of cases. Gram-negative bacteria (GNB) predominated (82.80%), with *Escherichia coli* being the most frequently isolated pathogen (45.70%). Among Enterobacteriaceae, 43.60% were extended-spectrum beta-lactamase (ESBL) producers, and

3.27% of GNB were carbapenem-resistant. Multivariate Poisson regression identified the Charlson Comorbidity Index (RR 1.11; 95% CI 1.02–1.21), sepsis (RR 2.26; 95% CI 1.19–4.29), septic shock (RR 2.74; 95% CI 1.58–4.76), and respiratory failure (RR 1.66; 95% CI 1.05–2.62) as independent factors associated with increased 28-day mortality. A urinary source of infection was found to be associated with less mortality (RR 0.38; 95% CI 0.19–0.77).

**Conclusion**: At this cancer referral center, one-third of patients with bacteremia died within 28 days. Mortality was primarily caused by the severity of infection and specific comorbidities, rather than antimicrobial resistance.

# Introduction

Bloodstream infections (BSI) are among the most serious and common complications in cancer patients. It is estimated that 10% to 38% of oncology patients develop BSI during their illness [1, 2]. This complication occurs in both solid and hematologic malignancies, with the risk being higher in the latter [3, 4]. Several host factors predispose cancer patients to BSI, including tumor-related factors such as physical obstruction from solid tumors, disruption of cutaneous and mucosal barriers, and immunosuppression caused by the malignancy. Additionally, treatment-related factors like chemotherapy, hematopoietic stem cell transplantation, neutropenia, corticosteroid therapy, or the use of invasive devices contribute to the risk [1, 5-7]. The most frequently isolated agents in cancer-associated bacteremia are Gram-negative bacilli, particularly *Escherichia coli* and *Klebsiella pneumoniae*, although less common Gram-positive bacteria (GPB) also remain significant causes [2,

8].

Despite advances in care, mortality associated with BSI among cancer patients remains high. Prior studies have reported 30-day mortality rates ranging from 17% to 32% [3, 4, 9]. Factors most strongly linked to mortality primarily relate to cancer status and the severity of clinical presentation [2, 10, 11]. Additionally, advanced age, corticosteroid use, and polymicrobial infection are recognized predictors of mortality [12, 13]. Inadequate empirical antibiotic therapy has also been identified as a marker for poorer outcomes in this group [11, 14]. Early mortality, occurring within

48–72 hours of diagnosis, is strongly associated with delays in initiating active antimicrobial therapy [2, 15]. The role of antimicrobial resistance in driving mortality among these patients remains unclear. While some observational studies have found a direct association between multidrugresistant organisms (MDROs) and increased mortality [12], this finding is not consistent across all cohorts of cancer patients with bacteremia [4, 16].

Few observational studies investigating bacteremia in cancer patients have been conducted in Latin America. Reported 30-day mortality rates are 17.5% in Argentina, 22% in Mexico, and 25.6% in Colombia [2, 3, 4]. Most research is restricted to specific cancer subgroups, limiting generalizability to broader settings. To our knowledge, no indexed studies have evaluated mortality and its risk factors in bacteremia among our national cancer population. This underscores the need for comprehensive data reflecting local epidemiology and patient characteristics, which is essential for filling knowledge gaps and guiding prevention and treatment strategies within our community. This study aimed to assess 28-day all-cause mortality and its associated factors among cancer patients with bacteremia at a national referral cancer center in Lima.

# Materials and methods

## Study design

A retrospective study was conducted at a single cancer referral center in Lima, Peru. From July 2020 through June 2024, all positive blood culture results were collected. Hospitalized adult patients with any malignancy who experienced their first episode of bacteremia were included. An episode was defined by at least one positive peripheral blood culture. For organisms listed as common commensals (National Healthcare Safety Network), the same organism had to be isolated from at least two separate cultures along with clinical signs of infection (fever, chills, or hypotension) to be considered accurate bacteremia [17, 18]. Individuals who were lost to follow-up after 28 days or transferred to another institution were excluded. Additionally, patients with synchronous neoplasia or without histopathological confirmation of cancer were not included in the study.

## Definitions

Sepsis and septic shock were diagnosed based on the criteria outlined by the third international consensus for sepsis and septic shock (Sepsis-3) [19]. The Sequential Organ Failure Assessment Score (SOFA) was calculated using clinical and laboratory data obtained closest to the time of culture collection. Empirical therapy was deemed adequate if at least one antibiotic with *in vitro* activity was started before susceptibility results were available. Bacteremia was classified as primary if no secondary sources were identified. It was considered persistent if the same organism was isolated again in a follow-up peripheral blood culture during the same hospital stay. An episode of bacteremia was classified as polymicrobial when two or more bacteria were isolated within the same 72-hour period [11]. The site of acquisition was categorized as nosocomial, community acquired, or healthcare associated. Nosocomial bacteremia included patients hospitalized for ≥48 hours in a general ward or intensive care unit before culture collection. Healthcare-associated bacteremia involved patients with any of the following within the past 90 days: hospitalization, intravenous chemotherapy, antibiotic treatment, hemodialysis, wound care, residence in a nursing home or assisted living facility, or the presence of an invasive device within the last 30 days before culture sampling [20]. Advanced cancer was defined as any malignant neoplasm with locally advanced or metastatic disease at the time of study. Active cancer was identified if the patient met any of these criteria: systemic treatment within the previous 6 months, recent tumor resection, end stage palliative care, recent diagnosis, or evidence of disease progression or recurrence. Immunosuppressive therapy was defined as patients who received any of the following within 30 days before blood culture: systemic chemotherapy, corticosteroids, monoclonal antibodies, tyrosine kinase inhibitors, or immunotherapy. Past antibiotic use was defined as having received any dose of antibiotics in the previous 30 days. Prior hospitalization meant patients who had been hospitalized for at least 48 hours within the last three months before the bacteremia episode. Comorbidities were assessed with the Charlson Comorbidity Index (CCI), a validated score that estimates 10-year survival probability [21].

## Microbiology

Multidrug-resistant Gram-negative bacteria (GNB) were defined as non-susceptible to at least one antibiotic in three or more different classes [22]. *Stenotrophomonas maltophilia* was included in this group due to its inherent resistance traits. Strains resistant to all tested antibiotic classes, except one or two, were categorized as extremely resistant GNB [22]. Difficult-to-treat resistant (DTR) GNB were those that show resistance or intermediate susceptibility to every antibiotic in three specific classes: beta-lactams, carbapenems, and fluoroquinolones [23]. The following Enterobacteriaceae species were considered AmpC cephalosporinase hyperproducers: *Enterobacter cloacae*, *Klebsiella aerogenes* and *Citrobacter freundii* [24]. Species at risk of AmpC hyperproduction that exhibited resistance to third-generation cephalosporins but susceptibility to cefepime were considered constitutive AmpC cephalosporinase hyperproducers [24]. Extended-spectrum beta-lactamase (ESBL) producers were identified by non-susceptibility to either ceftazidime, cefotaxime, ceftriaxone, or cefepime.

## Procedures

Laboratory technicians collected blood samples from the institution. Four peripheral samples, each with 5-10 mL of blood, were drawn for two blood culture sets. Each set contained two bottles with culture media—one for anaerobes and one for aerobes. The growth media used were BACT/ALERT®

SA Standard Aerobic (bioMérieux, France) and BACT/ALERT® SN Standard Anaerobic (bioMérieux, France), both containing 40 mL of Supplemented Tryptic Soy Broth. Samples were processed using the BACT/ALERT VIRTUO microbial detection system (bioMérieux, France), which employs a colorimetric method for growth detection. Positive samples were subsequently incubated for 18 to 24 hours in conventional growth media for further testing. Bacterial identification and susceptibility testing were performed using the automated VITEK 2 COMPACT system (bioMérieux, France), which utilizes cards tailored to the specific pathogen type. Susceptibility results were interpreted according to CLSI guidelines. Carbapenemases were detected via chromatographic tests: RAPIDEC® CARBA NP (bioMérieux, France) or OKNVI RESIST-5 (Coris BioConcept, Belgium).

## Statistical analysis

Bivariate analysis was conducted for both the primary and secondary outcomes. Chi-square test or

Fisher’s exact test was used for categorical variables. After assessing normality with the ShapiroWilk test, non-categorical variables were analyzed using the t-test or the Mann-Whitney U test. Multivariate analysis was also performed for both the primary and secondary outcomes. Variables included in the multivariate model were those with a p-value less than 0.20 and/or those deemed relevant by the investigators. The Poisson regression model was employed for both multivariate analyses. All variables were considered statistically significant at a p-value of less than 0.05.

## Ethical aspects

The project was approved by the Institutional Research Ethics Committee of Universidad Peruana Cayetano Heredia and Clínica Oncosalud. No access to patients' personal data was granted, as all information was coded for the study, ensuring that the authors had no access to participants’ identifying details. Data were collected retrospectively between August 1st and December 15th, 2024. Access to the database was restricted to the principal investigators.

# Results

## Study population

A total of 490 positive blood cultures were collected between July 2020 and June 2024. Of these, 61 were deemed contaminants, 39 were from non-hospitalized patients, 76 involved recurrent episodes of bacteremia, and 21 were excluded for other reasons. The study included 293 patients experiencing their first episode of bacteremia (Fig 1).

**Fig 1. Flowchart of patient selection.**

## Clinical and demographic characteristics

Solid tumors represented the most common underlying malignancy, accounting for 84.3% of cases. Most patients (91.81%) had active disease, and 80.73% had been diagnosed with advanced-stage cancer at the time of infection. At admission, 49.83% of patients presented with sepsis, while 33.79% experienced septic shock. Additionally, antibiotic use in the previous month was documented for 46.76% of cases, and 67.92% of patients had experienced a recent hospitalization.

Other clinical and demographic characteristics are summarized in Table 1.

### Table 1. Clinical and demographic characteristics of 293 patients with bacteremia*

**Characteristic**

**Total (n, %)**

| Age, years, mean + SD^†^ | 65.42 ± 15.3 | | |
| --- | --- | --- | --- |
| Female sex | 158 (53.92) | | |
| Charlson Comorbidity Index, mean + SD^†^ | 7.01 ± 2.81 | | |
| Hematologic malignancy | 46 (15.70) | | |
| Solid tumors | 247 (84.30) | | |
| Active cancer | 269 (91.81) | | |
| Advanced neoplasm, n = 275 | 222 (80.73) | | |
| Empirical antibiotic therapy | 285 (97.27) | | |
| Adequate empirical antibiotic therapy, n = 285 | 230 (80.70) | | |
| Sepsis | 146 (49.83) | | |
| Septic shock | 99 (33.79) | | |
| Respiratory failure on admission | 35 (11.95) | | |
| Severe neutropenia, n = 287 | | 29 (10.10) |  |
| Prior major surgery | | 95 (32.42) |  |
| Immunosuppressive therapy | | 129 (44.03) |  |
| Prior antibiotic use | | 137 (46.76) |  |
| Prior hospitalization | | 199 (67.92) |  |
| Central venous catheter | | 50 (17.06) |  |
| Port-a-Cath | | 102 (34.81) |  |

*Calculated on 293 patients unless otherwise specified. Values are number (%) unless otherwise noted.

†SD: standard deviation

Meropenem was the most frequently used empiric antibiotic (59.04%) and was significantly associated with higher rates of appropriate empiric therapy. For further information, refer to S1 and S2 Tables.

## Bacteremia Features and Microbiological Findings

The characteristics of bacteremia are listed in Table 2. Secondary bacteremia accounted for 53.92%, with urinary tract infections being the most common source at 23.89%. Nine cases (3.07%) were polymicrobial: five exclusively involved GNB, and four involved both GPB and GNB. Persistent bacteremia was assessed in 181 patients (61.77%) and was present in 17 (9.39%).

### Table 2. Bacteremia characteristics*

**Total (n,**

#### Characteristic

**%)**

| Bacteremia source |  |
| --- | --- |
| Primary | 135 (46.08) |
| Secondary | 158 (53.92) |
| Urinary | 70 (23.89) |
| Biliary | 34 (11.60) |
| Abdominal | 20 (6.83) |
| Respiratory | 19 (6.48) |
| Skin and soft tissue | 9 (3.07) |
| Other | 6 (2.05) |
| Place of bacteremia acquisition |  |
| Community | 41 (13.99) |
| Nosocomial | 48 (16.38 |
| Healthcare-associated | 204 (69.62) |
| Polymicrobial bacteremia | 9 (3.07) |
| Persistent bacteremia, n = 181 | 17 (9.39) |

*Calculated on 293 patients unless otherwise specified. Values are number (%) unless otherwise noted.

A total of 302 isolates were retrieved, with GNB predominating (82.78%). The most frequently isolated species was *Escherichia coli* (45.7%), followed by *Klebsiella pneumoniae* (14.24%). The different bacterial species isolated are shown in Table 3. Among GPB, Coagulase-negative

*Staphylococci* (CoNS) were the most common (6.95%), with *Staphylococcus epidermidis* prevailing (S3 Table).

#### Table 3. Distribution of bacterial isolates*

**Total (n, %)**

**Classification**

Gram

-

positive

52 (17.22)

| Coagulase-negative *staphylococci* | 21 (6.95) | | |
| --- | --- | --- | --- |
| *Staphylococcus aureus* | 12 (3.97) | | |
| *Enterococcus faecalis* | 11 (3.64) | | |
| *Streptococcus pyogenes* | 2 (0.66) | | |
| *Streptococcus anginosus* | 2 (0.66) | | |
| *Streptococcus agalactiae* | 1 (0.33) | | |
| *Streptococcus constellatus* | 1 (0.33) | | |
| *Enterococcus faecium* | 1 (0.33) | | |
| *Listeria monocytogenes* | 1 (0.33) | | |
| Gram-negative | 250 (82.78) | | |
| Fermenters | 215 (71.19) | | |
| *Escherichia coli* | 138 (45.70) | | |
| *Klebsiella pneumoniae* | | 43 (14.24) |  |
| *Serratia marcescens* | | 9 (2.98) |  |
| *Enterobacter cloacae* | | 8 (2.65) |  |
| *Proteus mirabilis* | | 4 (1.33) |  |
| *Salmonella enterica* | | 4 (1.33) |  |
| Other | | 9 (2.98) |  |
| Non-fermenters | | 29 (9.60) |  |
| *Pseudomonas aeruginosa* | | 20 (6.62) |  |
| *Acinetobacter baumannii* | | 4 (1.33) |  |
| *Stenotrophomonas maltophilia* | | 3 (0.99) |  |
| Other | | 2 (0.66) |  |
| Anaerobes | | 6 (1.99) |  |
| *Bacteroides sp.* | | 6 (1.99) |  |

*Calculated on 302 bacterial isolates unless otherwise specified. Values are number (%) unless otherwise noted.

*Acinetobacter baumannii* bacteremia had the highest mortality rate among all pathogens (75%), followed by CoNS (61.9%) and *P. aeruginosa* (40%). CoNS were the only species with a statistically significant higher mortality rate at 28 days compared to other pathogens (61.90% vs. 29.78%, p=0.00). 28-day mortality rates by bacterial species are shown in S4 Table.

## Antimicrobial Resistance Phenotypes

Resistance phenotypes are presented in Table 4. BSI caused by GNB showed a higher rate of antimicrobial resistance (AMR) compared to those caused by GPB (66.12% vs. 45.83%, p < 0.05). ESBL-mediated resistance to third-generation cephalosporins was the most common resistant phenotype in this cohort. About one-third of all bacteremia episodes were caused by ESBL producing Enterobacteriaceae. Among all Enterobacteriaceae isolates, 43.6% were ESBL producers. For *E. coli* and *K. pneumoniae,* the proportions were higher, with 51.45% and 51.16% ESBL-positive isolates, respectively. Fatality rates did not differ between ESBL-positive and ESBL-negative

Enterobacteriaceae bacteremia (see S5 Table). Only eight Gram-negative isolates were carbapenem-resistant, with a nonsignificant higher mortality rate (37.5%) compared to susceptible strains. All *Acinetobacter baumannii* isolates were carbapenem-resistant; Class D carbapenemases (OXA-23, OXA-24, OXA-25, and OXA-58) were detected in two, both from patients with intensive care unit (ICU)-acquired bacteremia. Among 20 *Pseudomonas aeruginosa* isolates, two were carbapenem-resistant: one produced a Class B carbapenemase (metallo-β-lactamase), and the other one showed probable resistance due to loss of the OprD2 porin or overproduction of efflux pumps, based on susceptibility patterns. The only two carbapenem-resistant *K. pneumoniae* strains produced OXA-48 carbapenemase. Among Gram-positive isolates, the most common resistant phenotype was Methicillin-resistant CoNS (MR-CoNS) (15/21). All *Staphylococcus aureus* strains were methicillin-susceptible, and no vancomycin-resistant GPB were identified.

### Table 4. Antimicrobial Resistance Profiles*

| **Phenotype** | **Total (n, %)** |
| --- | --- |
| Gram-negative bacteria | 245 (83.62) |
| Carbapenem-resistant, n = 245 | 8 (3.27) |
| Multidrug resistant, n = 245 | 100 (40.82) |
| Extensively drug resistant, n = 245 | 34 (13.88) |
| Difficult to treat resistance, n = 245 | 5 (2.04) |
| Extended spectrum β lactamase producing Enterobacteriaceae, n = 211 | 92 (43.60) |
| AmpC cephalosporinase-hyperproducing Enterobacteriaceae, n = 211 | 24 (11.37) |
| Constitutive AmpC cephalosporinase hyperproducers, n = 211 | 3 (1.42) |
| Gram positive bacteria | 52 (17.75) |
| *Staphylococcus aureus* | 12 (4.10) |
| Methicillin-resistant, n = 12 | 0 (0) |
| Coagulase negative *Staphylococcus* | 21 (7.17) |
| Methicillin-resistant, n = 21 | 15 (71.43) |
| *Enterococcus sp.* | 12 (4.10) |
| Ampicillin-resistant | 1 (8.33) |

*Calculated on 293 patients unless otherwise specified. Values are number (%) unless otherwise noted.

Total of Enterobacteriaceae (n) = 211

222

## 223 Clinical Outcomes

224 The 28-day all-cause mortality rate was 32.08%. The 48-hour and 90-day all-cause mortality rates
225 were 9.22% and 48.63%, respectively; 20.82% of patients were admitted to the ICU within 48 hours
226 of blood culture sampling. The clinical outcomes are shown in Table 5.

227

**Table 5. Clinical Outcomes***

| **Outcome** | **Total (n)** | **Percentage (%)** | **95% CI**^¶^ |
| --- | --- | --- | --- |
| All-cause mortality  48-hour | 27 | 9.22 | [6.41–13.08] |
| 28-day | 94 | 32.08 | [27.00–37.63] |
| 90-day^†^ | 142 | 48.63 | [42.80–54.17] |
| ICU admission within 48 hours | 61 | 20.82 | [16.56–25.83] |
| Readmission^‡^ | 112 | 56.28 | [32.85–43.91] |
| Bacteremia-associated | 20 | 17.86 | [11.78–26.25] |
| Length of stay, days, median with IR^§^  Total length of stay | 12 | [8-20] |  |
| Length of stay due to bacteremia | 10 | [6-17] |  |

*Calculated on 293 patients unless otherwise specified. Values are number (%) unless otherwise noted.

^†^n = 292, 1 lost to follow-up

^‡^n = 199, deceased patients at 28 days were not included

^§^IR = interquartile ranges

^¶^CI = confidence interval

228

## 229 Factors Associated with Mortality

230 Table 6 summarizes the bivariate and multivariate analyses for 28-day all-cause mortality.
231 Independent predictors of mortality included a higher CCI, the presence of sepsis and septic shock 232	on admission, and respiratory failure. Septic shock had the strongest association with mortality (RR,
233 2.74; 95% CI, 1.58-4.76; p < 0.0001). Among CCI components, heart failure and solid tumors
234 (localized or metastatic) were significantly associated with increased risk (S6 Table). Each additional
235 CCI point was associated with an 11% increase in mortality. The urinary source was found to 236 decrease the risk of mortality (RR 0.38; 95% CI, 0.19-0.77; p = 0.007). No specific resistant phenotype 237 was independently associated with mortality.

238

### Table 6. Bivariate and multivariate analysis of factors associated with 28 day all cause mortality*

|  | **28 day-all-cause mortality** | | | **Bivariat e** | **Multivariate** | | |
| --- | --- | --- | --- | --- | --- | --- | --- |
| **Characteristics** | **Alive (%) Deceased (%)** | | | **p-value** | **RR**^§^ **[95% CI**^¶^**] p-value** | | |
| Age, year, mean ± SD^†^ | | 64.70 ± 15.12 | 66.96 ± 15.67 | 0.239 | 1.00 [0.99-1.02] | 0.691 |  |
| Female sex | | 103 (51.76) | 55 (58.51) | 0.279 | 1.42 [0.93-2.16] | 0.106 |  |
| Charlson Comorbidity Index, mean ± SD^†^ | | 6.61 ± 2.80 | 7.87 ± 2.69 | 0.000 | 1.11 [1.02-1.21] | 0.015 |  |
| Urinary source | | 61 (30.65) | 9 (9.57) | 0.000 | 0.38 [0.19-0.77] | 0.007 |  |
| Respiratory failure | | 33 (16.58) | 50 (53.19) | 0.000 | 1.66 [1.05-2.62] | 0.020 |  |
| Sepsis | | 74 (37.19) | 72 (76.60) | 0.000 | 2.26 [1.19-4.29] | 0.010 |  |
| Septic shock | | 46 (23.12) | 53 (56.38) | 0.000 | 2.74 [1.58-4.76] | 0.000 |  |

*Calculated on 293 patients unless otherwise specified. Values are number (%) unless otherwise noted. ^†^SD: standard deviation; ^§^RR: relative risk; ^¶^CI: confidence interval

239

240 Early (48-hour) mortality predictors are shown in Table 7. Appropriate empiric antibiotic therapy
241 was associated with a significantly lower risk of early death (RR 0.32; 95% CI, 0.14-0.74; p = 0.007). 242 Conversely, primary BSI (RR 4.35; 95% CI, 1.59-11.91; p = 0.004), sepsis (RR, 14.87; 95% CI, 1.64243 134.29, p = 0.016), and septic shock (RR 41.45; 95% CI, 5.52-311; p = 0.000) were significantly

244	associated with early mortality.

245

### Table 7. Bivariate and multivariate analysis of factors associated with 48 hour all cause mortality*

|  | **48-hour all-cause mortality** | | | **Bivariat e** | **Multivariate** |  | |
| --- | --- | --- | --- | --- | --- | --- | --- |
| **Characteristics** | **Alive (%) Deceased (%)** | | | **p-value** | **RR**^§^ **[95% CI]** | **p-value** | |
| Age, year, mean ± SD^†^ | | 65.66 ± 15.33 | 63 ± 15.08 | 0.455 | 1.00 [0.98-1.03] | 0.571 |  |
| Female sex | | 142 (53.38) | 16 (59.26) | 0.559 | 1.35 [0.60-3.03] | 0.461 |  |
| Adequate empiric antibiotic therapy | | 214 (80.45) | 16 (59.26) | 0.011 | 0.32 [0.14-0.74] | 0.007 |  |
| Primary source of bacteremia | | 113 (42.48) | 22 (81.48) | 0.000 | 4.35 [1.59-11.91] | 0.004 |  |
| Sepsis | | 43 (16.17) | 4 (14.81) | 0.000 | 14.87 [1.64-134.29] | 0.016 |  |
| Septic shock | | 77 (28.95) | 22 (81.48) | 0.000 | 41.45 [5.52-311.00] | 0.000 |  |

**Calculated on 293 patients unless otherwise specified. Values are number (%) unless otherwise noted.* †SD: standard deviation; §RR: relative risk; CI: confidence interval

246

247

# 248 Discussion

In this cancer referral center, the 28-day all-cause mortality rate among patients who developed bacteremia was 32.08%. The primary independent factors associated with mortality were infection severity and comorbidities. ESBL-producing Enterobacteriaceae were the most common resistant pathogens, responsible for nearly half of the bacteremia cases. Contrary to expectations, antibiotic resistance was not independently linked to increased mortality.

The observed mortality rate in our cohort is among the highest reported so far for cancer patients with bacteremia. Previous studies have shown mortality rates ranging from 14.9% to 32%, depending on the predominant type of malignancy in each cohort [2, 4, 10, 16, 25]. When compared to cohorts from the same geographic area, our center’s mortality rate remains the highest [2-4, 26]. This may be partly due to the poor baseline prognosis of our cohort: we included a larger proportion of elderly patients, and the CCI score was significantly higher, even though the proportion of individuals with active or advanced cancer was similar to that of other cohorts [2, 4]. These findings highlight the unfavorable baseline expected survival of the patients included in our study.

The pre-infectious prognosis determined mortality in this cohort, as it was found to be independently associated with higher mortality rates. Comorbidities, including cancer, appear partially decisive in outcomes during a bacteremia episode. A higher CCI was independently linked to increased mortality, in agreement with Ha YE et al., who reported this association in cancer patients with *E. coli* bacteremia [25]. When analyzing the CCI components, we found statistically significant differences in 28-day survival rates between patients with local and metastatic neoplasms. Cancer burden has consistently been identified as a marker for higher mortality in bacteremia episodes [2-4, 10, 16]. Likewise, infection severity was a fundamental predictor of fatality. Septic shock at presentation was the strongest predictor of mortality in our cohort. Several studies have consistently identified sepsis and septic shock as independent variables associated with higher mortality [3, 9-11, 25, 27]. Our findings repeatedly emphasize the importance of individual baseline status in outcomes during invasive infection, as sepsis is known to result from an aberrant host immune response to infection [28]. Additionally, respiratory failure was independently linked to higher mortality rates, although no studies have specifically reported this association. Ha YE et al. noted that the need for mechanical ventilation predicted higher mortality [25]. In our cohort, respiratory failure could have resulted from either cancer progression or multiorgan failure caused by severe infection. This highlights the importance of comorbidities and infection severity in cancer patients with bacteremia.

Unlike previous reports, no association between antimicrobial resistance and mortality was found at this center. This may be partly attributable to the adequate rate of appropriate empirical therapy, particularly the frequent use of carbapenems, since ESBL-producing Enterobacteriaceae were the predominant antimicrobial-resistant phenotype in our cohort. Ha YE et al. demonstrated that ESBL presence was associated with increased mortality in cancer patients with bacteremia [25]. Similarly, Gudiol et al. reported higher mortality rates with Gram-negative MDR organisms [29]. While carbapenem-resistant GNB showed higher mortality rates than susceptible strains, this was not identified as an independent risk factor for mortality in the multivariate analysis. This finding has also been observed previously in hematologic cancer patients with bacteremia [3]. A type II error might account for the lack of association, possibly due to the low incidence of carbapenem-resistant strains at this center.

Antimicrobial resistance rates at our center generally align with the latest national data. ESBLproducing Enterobacteriaceae were the dominant AMR phenotype in our cohort and are recognized as a national issue. A multicenter study by Krapp et al. across various regions of Peru found a concerning 59.2% of third-generation cephalosporin-resistant GNB isolated from blood samples in public hospitals [30]. The lower ESBL-positive rate observed in our cohort may be due to a more robust infection control program, as it was conducted at a private center with economic advantages.

We identified eight carbapenem-resistant GNB, representing 3.27% of the total an even lower proportion compared to other regional studies [3, 4, 30]. No carbapenem-resistant *E. coli* were detected, which remains a rare strain in Peru [30]. Notably, no Methicillin-resistant *Staphylococcus aureus* (MRSA) bacteremia cases were identified, contrasting with previous local data showing a 36.1% prevalence [31]. Overall, these lower AMR rates likely reflect differences in resources and infection control practices between the private and public sectors in Peru. The lack of optimal antimicrobial stewardship programs continues to challenge our public hospitals.

The main limitation of this study is its retrospective design. The data analyzed were collected from a single center, reducing external validity. Additionally, the study period limited the amount of available data, thereby affecting the statistical power for certain variables. This constrained the ability to detect differences for some factors, particularly resistant strains that appeared infrequently. However, this is the first study to evaluate the impact of bacteremia in individuals with cancer in Peru. Globally, our findings contribute to the existing evidence on this common infectious complication in people with cancer.

# Conclusions

In this group of individuals with cancer and bacteremia, about one-third of patients died within 28 days of diagnosis. The severity of the infection and comorbidities mainly influenced mortality.

Antimicrobial-resistant bacteria were not linked to worse outcomes at this center.

# References

1. See I, Freifeld AG, Magill SS. Causative organisms and associated antimicrobial resistance in healthcare-associated, central line–associated bloodstream infections from oncology settings, 2009–2012. Clin Infect Dis. 2016 May 15;62(10):1203-9
2. Islas-Muñoz B, Volkow-Fernández P, Ibanes-Gutiérrez C, et al. Bloodstream infections in cancer patients: risk factors associated with mortality. Int J Infect Dis. 2018 Jun;71:59-64.
3. Herrera F, Torres D, Laborde A, Jordán R, Berruezo L, Roccia Rossi I, et al. Epidemiology of Bacteremia in Patients with Hematological Malignancies and Hematopoietic Stem Cell

Transplantation and the Impact of Antibiotic Resistance on Mortality: Data from a Multicenter Study in Argentina. Pathogens. 2024;13(11).

4. Cruz-Vargas SA, García-Muñoz L, Cuervo-Maldonado SI, Álvarez-Moreno CA, SaavedraTrujillo CH, Álvarez-Rodríguez JC, et al. Molecular and Clinical Data of Antimicrobial Resistance in Microorganisms Producing Bacteremia in a Multicentric Cohort of Patients with Cancer in a Latin American Country. Microorganisms. 2023 Feb 1;11(2).
5. Gudiol C, Albasanz-Puig A, Cuervo G, Carratalà J. Understanding and Managing Sepsis in

Patients With Cancer in the Era of Antimicrobial Resistance. Front Med. 2021;8(March).

6. Rolston KVI. Infections in Cancer Patients with Solid Tumors: A Review. Infect Dis Ther.

2017;6(1):69–83.

7. Samonis G, Vardakas KZ, Maraki S, Tansarli GS, Dimopoulou D, Kofteridis DP, et al. A prospective study of characteristics and outcomes of bacteremia in patients with solid organ or hematologic malignancies. Support Care Cancer. 2013;21(9):2521–6.
8. Amanati A, Sajedianfard S, Khajeh S, Ghasempour S, Mehrangiz S, Nematolahi S, et al. Bloodstream infections in adult patients with malignancy: epidemiology, microbiology, and risk factors associated with mortality and multi-drug resistance. BMC Infect Dis.

2021;21(1):1–14.

9. Kang CI, Song JH, Chung DR, Peck KR, Yeom JS, Son JS, et al. Bloodstream infections in adult patients with cancer: Clinical features and pathogenic significance of Staphylococcus aureus bacteremia. Support Care Cancer. 2012;20(10):2371–8.
10. Marín M, Gudiol C, Garcia-Vidal C, Ardanuy C, Carratalà J. Bloodstream infections in patients with solid tumors: Epidemiology, antibiotic therapy, and outcomes in 528 episodes in a single cancer center. Med (United States) [Internet]. 2014 May;93(3):143–9. Available from: https://journals.lww.com/00005792-201405000-00003
11. Royo-Cebrecos C, Gudiol C, Ardanuy C, Pomares H, Calvo M, Carratalà J. A fresh look at polymicrobial bloodstream infection in cancer patients. PLoS One. 2017;12(10):1–12.
12. Park KH, Jung YJ, Lee HJ, Kim HJ, Maeng CH, Baek SK, et al. Impact of multidrug resistance on outcomes in hematologic cancer patients with bacterial bloodstream infections. Sci Rep. 2024 Dec 1;14(1).
13. Xue L, Zhu Y, Zong M, Jiao P, Fu J, Liang XM, et al. Clinical characteristics of bloodstream infections in adult patients with solid tumours and a nomogram for mortality prediction: a 5-year case-controlled retrospective study in a tertiary-level hospital. Front Cell Infect Microbiol. 2023;13(August):1–12.
14. Gudiol C, Tubau F, Calatayud L, Garcia-Vidal C, Cisnal M, Sánchez-Ortega I, et al. Bacteraemia due to multidrug-resistant gram-negative bacilli in cancer patients: Risk factors, antibiotic therapy and outcomes. J Antimicrob Chemother. 2011;66(3):657–63.
15. Tang Y, Wu X, Cheng Q, Li X. Inappropriate initial antimicrobial therapy for hematological malignancies patients with Gram-negative bloodstream infections. Infection [Internet]. 2020;48(1):109–16. Available from: <https://doi.org/10.1007/s15010-019-01370-x>
16. Park KH, Jung YJ, Lee HJ, Kim HJ, Maeng CH, Baek SK, et al. Impact of multidrug resistance on outcomes in hematologic cancer patients with bacterial bloodstream infections. Sci Rep.

2024 Dec 1;14(1).

17. Infectious Diseases Society of America (IDSA). Bloodstream Infection Event (Central LineAssociated Bloodstream Infection and Non-central Line-Associated Bloodstream Infection) [Internet]. 2024. Available from: https://www.idsociety.org
18. Centers for Disease Control and Prevention (CDC). NHSN Pathogen/Organism Resources [Internet]. Atlanta, GA: CDC; [cited 2025 Sep 14]. Available from:

[https://www.cdc.gov/nhsn/pathogens/index.html](https://www.cdc.gov/nhsn/pathogens/index.html?utm_source=chatgpt.com)

19. Singer M, Deutschman CS, Seymour C, Shankar-Hari M, Annane D, Bauer M, et al. The third international consensus definitions for sepsis and septic shock (Sepsis-3). JAMA.

2016;315(8):801-10.

20. Friedman ND, Kaye KS, Stout JE, McGarry SA, Trivette SL, Briggs JP, et al. Health care– associated bloodstream infections in adults: a reason to change the accepted definition of community-acquired infections. Ann Intern Med. 2002;137(10):791-7.
21. Charlson ME, Pompei P, Ales KL, MacKenzie CR. A new method of classifying prognostic comorbidity in longitudinal studies: development and validation. *J Chronic Dis.*

1987;40(5):373-83. doi:10.1016/0021-9681(87)90171-8.

22. Magiorakos AP, Srinivasan A, Carey RB, Carmeli Y, Falagas ME, Giske CG, et al. Multidrugresistant, extensively drug-resistant and pandrug-resistant bacteria: an international expert proposal for interim standard definitions for acquired resistance. Clin Microbiol Infect.

2012;18(3):268-81. doi:10.1111/j.1469-0691.2011.03570.x

23. Kadri SS, Adjemian J, Lai YL, Spaulding AB, Ricotta E, Prevots DR, et al. Difficult-to-treat resistance in Gram-negative bacteremia at 173 US hospitals: retrospective cohort analysis of prevalence, predictors, and outcome of resistance to all first-line agents. Clin Infect Dis.

2018;67(12):1803-14. doi:10.1093/cid/ciy378

24. Tamma PD, Heil EL, Justo JA, Mathers AJ, Satlin MJ, Bonomo RA, et al. Infectious Diseases Society of America 2024 guidance on the treatment of antimicrobial-resistant Gram-negative infections. Clin Infect Dis. 2024;79(1):1-56. doi:10.1093/cid/ciae403
25. Ha YE, Kang CI, Cha MK, Park SY, Wi YM, Chung DR, et al. Epidemiology and clinical outcomes of bloodstream infections caused by extended-spectrum β-lactamase-producing Escherichia coli in patients with cancer. Int J Antimicrob Agents. 2013;42(5):403-9.

doi:10.1016/j.ijantimicag.2013.06.021

26. Sierra J, Díaz MV, De Jesús García M, Finello M, Suasnabar DF, Richetta L, et al. Infecciones del torrente sanguíneo en pacientes oncológicos. Medicina (B Aires). 2020;80(4):329-38. Available from: [http://www.medicinabuenosaires.com/revistas/vol80-20/n4/329.pdf](http://www.medicinabuenosaires.com/revistas/vol80-20/n4/329.pdf?utm_source=chatgpt.com)
27. Antonio M, Gudiol C, Royo-Cebrecos C, Grillo S, Ardanuy C, Carratalà J. Current etiology, clinical features and outcomes of bacteremia in older patients with solid tumors. J Geriatr Oncol. 2018;9(4):286-90. doi:10.1016/j.jgo.2018.06.011
28. Meyer NJ, Prescott HC. Sepsis and septic shock. N Engl J Med. 2024 Dec 5;391(22):2133-46. doi:10.1056/NEJMra2403213
29. Gudiol C, Calatayud L, Garcia-Vidal C, Lora-Tamayo J, Cisnal M, Duarte R, et al. Bacteraemia due to extended-spectrum β-lactamase-producing *Escherichia coli* in cancer patients: clinical features, risk factors, molecular epidemiology and outcome. J Antimicrob Chemother.

2010;65(2):333-41. doi:10.1093/jac/dkp427

30. Krapp F, García C, Hinostroza N, Astocondor L, Rondon CR, Ingelbeen B, Alpaca-Salvador HA, Amaro C, et al. Prevalence of antimicrobial resistance in Gram-negative bacteria bloodstream infections in Peru and associated outcomes: VIRAPERU study. Am J Trop Med Hyg. 2023;109(5):1095-106. doi:10.4269/ajtmh.22-0556. PMID: 37722663; PMCID:

PMC10622474

31. García C, Hinostroza N, Gordillo V, Inchaustegui ML, Astocondor L, Chincha O, Alejos S, et al. Methicillin-resistant *Staphylococcus aureus* bloodstream infections in hospitalized patients in Peru. Am J Trop Med Hyg. 2023;109(5):1118-21. doi:10.4269/ajtmh.23-0054. PMID:

37722664; PMCID: PMC10622478

# Supporting information captions

**Supplemental Table 1.** Empiric antibiotic therapy and 28-day all-cause mortality bivariate analysis

**Supplemental Table 2.** Appropriate empirical therapy according to antibiotic usage

**Supplemental Table 3.** Coagulase-Negative staphylococci species

**Supplemental Table 4.** 28-day all-cause-mortality by bacterial species

**Supplemental Table 5.** Antimicrobial Resistance profiles and 28-day all-cause-mortality

**Supplemental Table 6.** 28-day all-cause mortality according to the Charlson Comorbidity Index components

Figure 1	[Click here to access/download;Figure;Fig 1.tiff](https://www2.cloud.editorialmanager.com/pone/download.aspx?id=42644592&guid=66d3e798-8e87-4b94-8ca5-a6acb7bd67da&scheme=1)

Supplemental tables

[Click here to access/download](https://www2.cloud.editorialmanager.com/pone/download.aspx?id=42644535&guid=b2004e24-394e-4af6-8d72-df53426f074c&scheme=1)

[Supporting Information S_file.pdf](https://www2.cloud.editorialmanager.com/pone/download.aspx?id=42644535&guid=b2004e24-394e-4af6-8d72-df53426f074c&scheme=1)
